# Impact of long-term use of oral nutritional supplement on nutritional adequacy, dietary diversity, food intake and growth of Filipino preschool children

**DOI:** 10.1017/jns.2016.6

**Published:** 2016-05-13

**Authors:** Dieu T. T. Huynh, Elvira Estorninos, Maria Rosario Capeding, Jeffery S. Oliver, Yen Ling Low, Francisco J. Rosales

**Affiliations:** 1 Abbott Laboratories, Abbott Nutrition Research & Development, Asia-Pacific Centre, Singapore; 2 Asian Hospital and Medical Centre, Manila, Philippines; 3 Abbott Laboratories, Scientific and Medical Affairs, Abbott Nutrition, Columbus, OH, USA

**Keywords:** Long-term supplementation, Oral nutritional supplementation, Nutritional adequacy, Dietary diversity, Longitudinal growth, DDS, dietary diversity score, FNRI, Food and Nutrition Research Institute, GEE, generalised estimating equation, HAP, height-for-age percentile, IDES, Individual Dietary Evaluation Software, ONS, oral nutritional supplementation, WHP, weight-for-height percentile

## Abstract

Nutrient deficiencies during childhood have adverse effects on child growth and health. In a single-arm 48-week long-term intervention, we previously reported the efficacy of oral nutritional supplementation (ONS) and dietary counselling on catch-up growth and growth maintenance in nutritionally at-risk Filipino children. The present analysis was done to assess the contributing effects of ONS to nutritional adequacy, dietary diversity, food intake and longitudinal growth. ONS (450 ml) was consumed daily providing 450 kcal (1880 kJ) and at least 50 % of micronutrient requirements among 200 children aged 3–4 years with weight-for-height percentiles between 5th and 25th (WHO Growth Standards). Weight, height and dietary intakes using 24-h food recalls were measured at baseline, and at weeks 4, 8, 16, 24, 32, 40 and 48. Nutrient adequacy and dietary diversity score (DDS) were calculated. Generalised estimating equations were used to assess the effects of total nutrient intakes, DDS, ONS compliance and sociodemographic factors on longitudinal growth. The percentages of children with adequate intake of energy, protein, Fe, Ca and some vitamins at each post-baseline visit were improved from baseline, reaching 100 % for most nutrients. DDS was also increased from baseline and reached significance from week 16 onwards (*P* < 0·01). Male children, total energy intake and parental employment status were associated with weight-for-height percentile gain (*P* < 0·05), whereas higher parental education level and ONS compliance were significantly associated with height-for-age percentile gain over time (*P* < 0·05). Long-term ONS intervention did not interfere with normal food intake and helped promote nutritional adequacy and growth of Filipino children.

Adequate and balanced energy, macronutrient and micronutrient intakes are vital for the physical and mental development of infants and children^(^^1^^)^. However, Filipino children have been reported to consume diets that are inadequate in some nutrients and energy. Dietary intake data using a single 24-h recall in a cohort of Filipino breast-fed infants and toddlers showed that intakes of energy and vitamins for all age groups were below the estimated needs due to the low nutrient densities of their diets^(^^2^^)^. In addition, more than 80 and 50 % of children aged 0–5 years had energy and protein consumption, respectively, not meeting the recommendation in the 2008 National Nutrition Survey^(^^3^^)^. According to the 2013 National Nutrition Survey, child undernutrition is still common in the Philippines in which 19·9 and 30·3 % of children aged 0–5 years were underweight and stunted, respectively^(^^4^^)^. Thus, there is a need to identify effective management approaches to improve the existing diet and improve child nutrition in this country.

Prevention and treatment of child undernutrition require diets providing adequate energy and essential nutrients to promote catch-up growth in weight and height, strengthen resistance to infection, and support normal mental, physical and metabolic development^(^^5^^)^. Counselling caregivers about the appropriate foods in adequate quantities is considered an integral part of treating child undernutrition^(^^6^^)^. However, there have been recognised challenges which may undermine its effectiveness in providing the nutrients required for improving growth in the setting of developing countries. In addition to the challenges in procuring dietary diversity and in consuming adequate amounts to meet nutrient intake recommendations, the commonly consumed foods in countries like South-East Asia countries, including the Philippines, such as cereals and legumes, are known to be relatively high in anti-nutrient content and have low nutrient bioavailability for some nutrients such as Fe, Zn and vitamin A^(^^2^^,^^7^^)^. Nutritional supplementation and food fortification are therefore recommended for achieving a desired nutrient density and nutrient adequacy to promote growth in children with undernutrition^(^^8^^)^. In fact, dietary counselling using family foods has been shown to be more effective when combined with oral nutritional supplementation (ONS) of macro- and micronutrients in improving nutritional status in nutritionally at-risk children when compared with dietary counselling alone^(^^9^^,^^10^^)^. Incorporating ONS in nutritional intervention strategy, therefore, may help overcome the shortcomings of dietary counselling relying solely on foods. We recently reported on a 48-week nutritional intervention that dietary counselling and the long-term use of ONS helped achieve catch-up growth and growth maintenance among Filipino preschoolers at risk of undernutrition^(^^11^^)^. We showed that weight-for-height percentiles (WHP) had the greatest increase in the first 4 weeks (*P* < 0·0001), and height-for-age percentiles (HAP) increased steadily over time and became significantly higher than baseline from week 24 onwards (*P* < 0·0001)^(^^11^^)^.

In the present study, we investigated the impact of the intervention on the nutritional adequacy and the role of nutrient intake in promoting improved longitudinal growth in nutritionally at-risk children. In addition, one common concern over the use of ONS is its potential displacement of normal food intake. Hence, we evaluated dietary diversity and food consumption while receiving the ONS using the collected dietary intake data over the study period.

## Materials and methods

### Study design and participants

Details of this study have been reported previously^(^^11^^)^. Briefly, this prospective, multicentre, single-arm study was designed to assess the compliance levels with nutritional intervention and its impact on growth. The study was conducted in the city of Muntinlupa, Philippines between October 2011 and October 2012. Clinically healthy children were eligible for inclusion if they were 36 to 48 months of age and at risk of undernutrition defined as WHP between the 5th and 25th percentile based on the WHO Growth Standards^(^^12^^)^. The exclusion criteria included a history of preterm delivery, birth weight <2500 or >4000 g, or a current or chronic infection (except for intestinal parasite infection), diarrhoea, acute and chronic hepatitis B or C, HIV or tuberculosis, or diagnosis of neoplastic diseases, renal, hepatic and cardiovascular diseases, or diagnosis of congenital or genetic disorder or infantile anorexia nervosa. Children who had an obese parent defined as measured or self-reported BMI ≥ 27·5 kg/m^2^ were also excluded to minimise the impact of genetic and family environmental influences on childhood overweight development^(^^13^^)^.

The study was approved by the Institutional Review Board and the Food and Drug Administration of the Philippines. A written informed consent was obtained from each child's parents or legal guardian. The study was performed in accordance with the ethical principles that had their origin in the Declaration of Helsinki (clinicaltrials.gov number NCT01658267).

### Intervention

The Recommended Energy and Nutrient Intakes for Filipino children aged 4 to 6 years^(^^14^^)^ and materials on good feeding practice from the WHO^(^^15^^)^ were used as guidelines for preparing the content of the dietary counselling. Three sessions on dietary counselling were administered by trained study physicians at baseline, and at weeks 4 and 8 post-baseline only. Parents were advised on food group selection, for which they were provided a list of foods with good protein quality from animal and vegetable sources, foods containing vegetable fat and locally available fatty fishes, staple foods, and also foods with dietary fibres such as beans, lentils and peas. In addition, some portion sizes of cooked foods from different food groups were given to aid parents or caregivers in estimating the adequate amounts to be consumed by the child. The methods for enhancing the child's eating behaviour with respect to mealtime and feeding environment, meal frequency, and limiting the consumption of sweetened foods and beverages were also given during the session. The ONS, commercially available (PediaSure^®^; Abbott Laboratories), was used to supplement the energy and protein content, and the essential nutrients that were previously reported to be lacking in the diet of Filipino children^(^^2^^,^^7^^)^. Parents were instructed to provide two servings of ONS to their child every day for 48 weeks. When given twice daily, the ONS provided 450 kcal (1880 kJ), 13·5 g high-quality protein, 17·7 g easily digested fat and 59·4 g carbohydrate and complete minerals and vitamins (450 ml in total) to meet about 30 and 50 % of the energy and micronutrient requirements, respectively^(^^11^^)^.

### Outcome assessments

The outcome measures included compliance with ONS consumption, changes in nutritional status as determined by weight-for-age, weight-for-height and height-for-age over the study period. Other outcomes included parental assessment of child's physical activity level at baseline and each post-baseline visit. The total energy, macronutrient and micronutrient intakes, the adequacy of energy and nutrient intakes according to the local recommendations, dietary diversity scores (DDS) and food intake at baseline and each post-baseline visit were analysed to understand the impact of the nutritional intervention on promoting the child's diet quality, thereby improving nutritional status as reported previously^(^^11^^)^.

### Compliance

Compliance with the study product was assessed from the product intake records that parents or caregivers were instructed to complete on a daily basis. The compliance was calculated by dividing the actual study product intake with the recommended intake over the study period and multiplying by 100.

### Anthropometric assessment

Anthropometric measurements were performed by study staff who had been trained on standardised methods of conducting the measurements. Weight was measured with light clothes and shoes and jackets removed, using electronic weighing scales (Tanita HD380) and recorded to the nearest 0·1 kg. Standing height was measured without shoes or hat, using a height measuring gauge (Seca 217) and recorded to the nearest 0·1 cm. Weight and height were measured at baseline and all post-baseline visits at weeks 4, 8, 16, 24, 32, 40 and 48. Weight-for-age, weight-for-height and height-for-age were expressed as sex–age-specific percentiles using the WHO Child Growth Standards.

### Dietary assessment

Dietary intake was collected using the 24-h food recall method by trained nutritionists from the Food and Nutrition Research Institute (FNRI) in the Philippines. Because the average dietary intake of the group using a single 24-h recall is suggested to be robust and unaffected by within-person variation^(^^16^^)^, we administered one 24-h food recall at baseline, taking into consideration reducing the respondent burden for completing the baseline evaluation prior to receiving the intervention. During the food recall interview at baseline, parents or the main caregiver were also instructed to use common household measures (cups, tablespoons, teaspoons, ruler, dimensions, etc.) to estimate the portion sizes and quantities of foods. One set of the household measures was provided to each child's parents to bring home for aiding the recall by telephone in the post-baseline visits. For all post-baseline visits, two 24-h food recalls for two non-consecutive days were done. The first recall was conducted in the weekend prior to the scheduled visit via telephone and the second recall was completed at the visit. The quality-control check for data accuracy and completeness was performed before conducting dietary intake data analysis using Individual Dietary Evaluation Software (IDES) developed by the FNRI. Nutrient intakes that are available in the Filipino food composition tables including protein, carbohydrate, fat, Ca, Fe, vitamin A, vitamin C, thiamin, riboflavin and niacin were calculated using IDES. Average energy and nutrients were computed from the two 24-h recalls for all post-baseline visits.

### Misreporters

Misreporters including under- and over-reporters were determined using the following methods. The BMR (MJ/d) was first calculated using Schofield's equations for children aged 3 to 10 years as follows^(^^17^^)^:

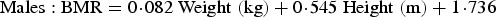


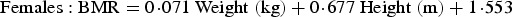


The BMR in kJ was converted into kcal using the conversion factor of 1 kcal for 4·185 kJ. The Goldberg cut-offs based on an assumed light physical activity level were then applied to classify the under-reporters and over-reporters. There are different proposed cut-offs for the ratios of total energy intake and BMR if the total energy intake was measured by one and two 24-h food recalls. For the baseline visit with one completed 24-h recall, under-reporters and over-reporters are defined as total energy intake/BMR <0·87 and >2·75, respectively. For all the post-baseline visits where two non-consecutive 24-h recall were completed, under-reporters and over-reporters are defined as total energy intake/BMR <0·96 and >2·49, respectively^(^^18^^)^. The misreporters identified at each visit were excluded from analyses on nutrient adequacy. They were also excluded from all visits for studying nutrient intake from food alone consumption over the study period.

### Assessment of nutrient adequacy

The probability approach and the estimated average requirement (EAR) cutpoint are recommended methods for evaluating the adequacy of nutrient intakes of population groups. However, these two methods require the EAR for nutrients that is not available for Filipino children. The alternative method using 77 % of the RDA as a cut-off value is employed for evaluating nutrient adequacy in this study^(^^19^^)^. The approximations for the mean nutrient requirements relied on the assumption that the recommended intakes for Filipino children aged 4 to 6 years estimates the mean requirement plus two standard deviations, with a CV of 15 %. Such an assumption yields a conservative estimate of nutrient inadequacy compared with a CV for the nutrient of 10 %^(^^19^^)^. The total energy intake was compared with the age-specific recommendation for energy intake, and the adequacy of nutrient intakes was determined by the intakes at or above 77 % cut-off of the recommended nutrient intakes for Filipino children aged 4 to 6 years^(^^14^^)^. The post-baseline time points at weeks 4, 32 and 48 are selected to present the nutrient adequacy during the catch-up growth period (week 4) and for the growth maintenance phase (weeks 32 and 48). In addition, the recommendation for the energy contribution of carbohydrates, fats and protein to the total energy intake for Filipino children such as 55–70, 20–30 and 10–15 %, respectively, was also used to estimate the percentage of children who consumed diets following the recommendation at baseline and each follow-up visit.

### Dietary diversity score

The DDS is a simple and quick indicator of nutrient adequacy and nutritional quality of the diet which can be used to assess the impact of the intervention on diet quality^(^^20^^)^. We used the method developed by the FAO^(^^20^^)^ to calculate the DDS based on the dietary intake data collected from the 24-h recalls. If at least one food or no foods under an appropriate food group was consumed the corresponding food group was assigned a score ‘1’ or ‘0’, respectively. The DDS will be the sum of the scores assigned to the different food groups. In total, there will be nine food groups (starchy staples; dark leafy vegetables; other vitamin A-rich fruits and vegetables; other fruits and vegetables; organ meat; meat and fish; eggs; legumes; nuts and seeds; and milk and milk products), and the sum of the scores can range from 0 to 9^(^^20^^)^. For all post-baseline visits, the DDS was averaged from the DDS calculated for the weekday and the weekend day recalls. Because all children received ONS (from the milk and milk product food group), the analysis of DDS was performed with and without this food group to evaluate if ONS contributed to the improved DDS at post-baseline visits.

### Energy-adjusted nutrient intakes as factors in modelling

Total energy and nutrient intakes from foods and ONS in all children were considered in modelling. Adjustment for total energy intake is recommended to control for confounding, reduce extraneous variation, and predict the effect of dietary intervention^(^^21^^)^. The energy-adjusted nutrient intakes were computed as the residuals from the regression model in which energy intake was the independent variable and absolute nutrient intake was the dependent variable, using the method of Willett & Stampfer^(^^21^^)^. Because of non-normal distribution, protein, Ca, Fe, vitamin A, thiamin, riboflavin and niacin were log-transformed while vitamin C was transformed using square root function, before their inclusion in the regression models. The total intake scores were next considered in the models of factors associated with growth as covariates.

### Physical activity

Parents were asked to rate their child's physical activity level over the last 24 h using visual analogue scales scoring 1 to 10 at baseline and all post-baseline visits. This method was used to rank the child's physical activity level rather than provide an objective measure of physical activity. Physical activity score at all visits was considered in the models of factors associated with growth.

### Statistical methods

All the statistical analyses were performed on the intent-to-treat population using SAS version 9.2. Descriptive results were summarised by means, medians and standard deviations. Categorical variables were summarised by number of subjects (*n*) and percentages (%). McNemar's test with step-down Bonferroni adjustment was used to compare the percentages between baseline and each post-baseline visit. Continuous variables were checked for normality. If a variable was found to be non-normal, the signed-rank test with step-down Bonferroni adjustment was used to examine the differences between two groups for continuous outcome variables with non-normal distribution. For assessing whether the energy and nutrient intakes from foods alone increased over the 48 weeks, the average of the regression slopes fitted for baseline and all post-baseline time points for individual subjects was tested for a difference from zero using data from normal reporters. *P* ≤ 0·05 and 0·05 < *P* ≤ 0·1 are considered significant and trend, respectively.

Generalised estimating equations (GEE) using autoregressive correlation structure were used to assess the longitudinal relationship between growth measured as WHP and HAP and total energy and nutrient intakes, while controlling for other factors collected at baseline and post-baseline visits over 48 weeks^(^^22^^)^. These factors included highest parental occupation and education, and the child's characteristics including age, sex and birth weight. Physical activity level was also included in modelling. The nutritional contribution of the ONS was determined based on its compliance expressed as percentage of actual consumption *v*. recommendation of 450 ml per d over the study period.

The selection of variables for inclusion into the GEE models was performed with the preliminary model in which the association between a single variable and the growth percentile development was examined. Factors with *P* < 0·1 from preliminary models and of established biological significance including age (months), sex and time (visit) were included in modelling. Only factors with *P* <0·1 and those with established biological importance remained in the final models. The adjusted estimates and the 95 % CI derived from the final GEE models are presented. The visit and sex interaction term was also included in the final models if any of the factor combinations was significant in the preliminary model.

## Results

The sociodemographic and anthropometric characteristics of the study subjects have been previously presented^(^^11^^)^. Briefly, 200 subjects were enrolled into the study from 224 screened subjects. Of the 200 enrolled subjects, one subject did not meet the eligibility criteria for age group and never consumed the study product, resulting in an intent-to-treat population of 199 subjects. The mean age of the study children was 41·2 months and 50 % were males. Males were heavier than females (*P* < 0·0001), while mean height was comparable between the two sexes. The mean WHP therefore was also significantly higher for males (*P* = 0·0228). In addition, the education level of the parents was high, with more than 90 % having attended high school and/or college or university. Furthermore, 98 % of fathers were employed, most of them working in the private sector, whereas most mothers were unemployed (data not shown).

### Misreporting

Using Schofield's equations and Goldberg cut-offs to classify misreporters (under- and over-reporters) at baseline and each post-baseline visit, we found that the percentage of over-reporters was increased from 6·0 % at baseline to 39·5 % at week 48, whereas under-reporting was not as prevalent as over-reporting with 4·5 % at baseline and 0·5 % at week 4 and week 24 (data not shown). The mean weight-for-age percentile, HAP and WHP were not statistically significantly different between the misreporters and those considered normal reporters at all visits, except for weight-for-age at week 48 where the normal reporters had higher weight-for-age percentile (*P* = 0·0421) (data not shown).

### Nutrient adequacy

**Fig. 1.**
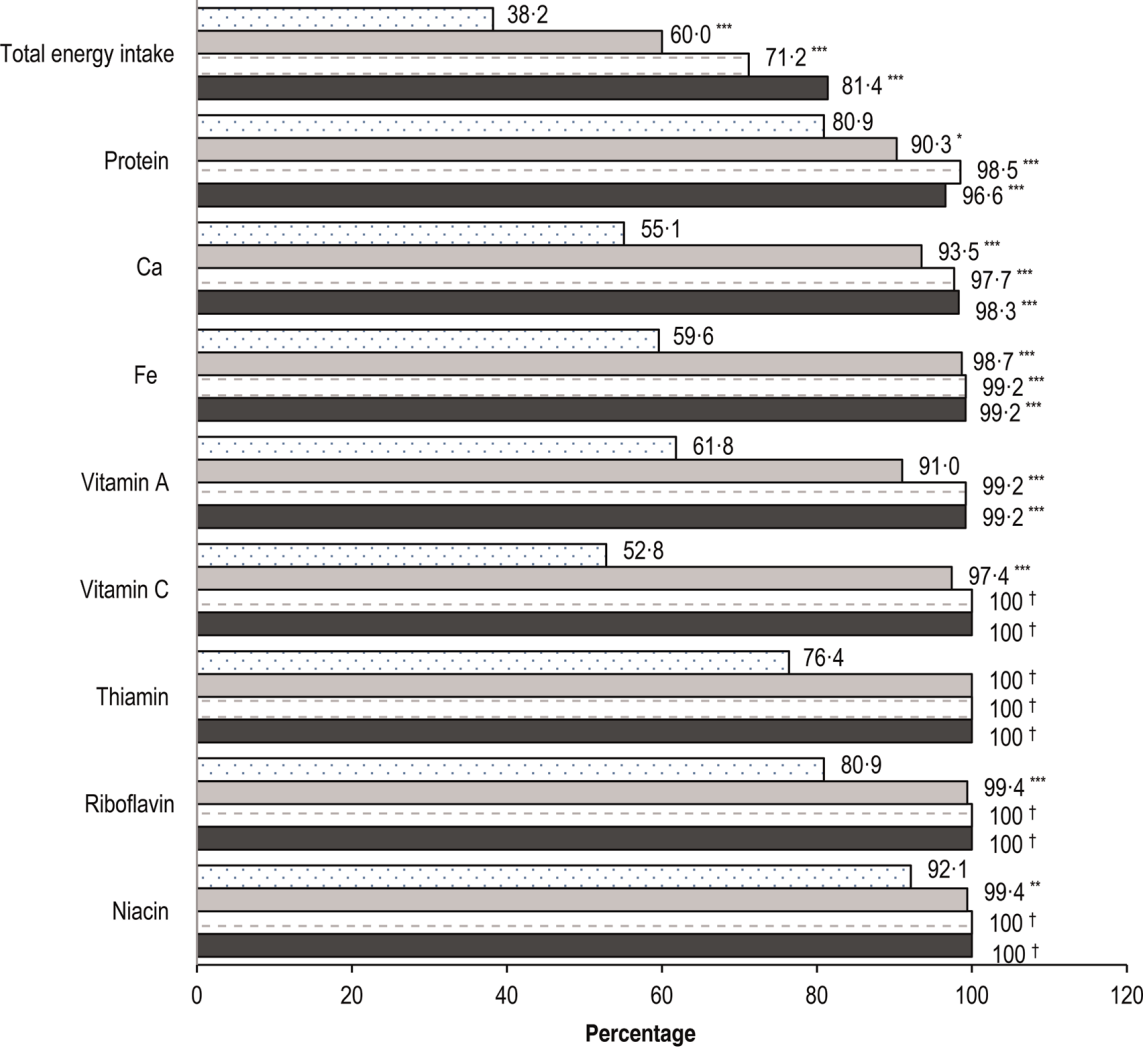
Percentage of children with adequate nutrient intakes at baseline (░), and at weeks 4 (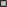), 32 (=) and 48 (■). *P* values are from McNemar's test and controlled for multiple comparisons using step-down Bonferroni adjustment: * *P* < 0·05, ** *P* < 0·01, *** *P* ≤ 0·0001. † McNemar's test could not be performed since all values were adequate.

Fig. 1 shows the percentage of children with adequate energy, protein, Ca, Fe, vitamin A, vitamin C, thiamin, riboflavin and niacin intakes at study baseline and at weeks 4, 32 and 48 post-baseline. Overall, one-third to two-thirds of children had adequate intake of energy, Ca, Fe, vitamin A and vitamin C before the intervention. The percentage of children with adequate intake of these nutrients was significantly increased within 4 weeks of the intervention (*P* < 0·0001), and remained significantly greater than baseline or reached 100 % for the remaining study period. The consumption of protein and some B vitamins including thiamin, riboflavin and niacin was adequate in at least three-quarters of children at baseline. The percentage of children with adequate intake of protein, thiamin, riboflavin and niacin was significantly greater than baseline or reached 100 % from week 4 onwards.

### Food group consumption pattern, dietary diversity score and compliance with recommendation on energy contribution from macronutrients

**Fig. 2.**
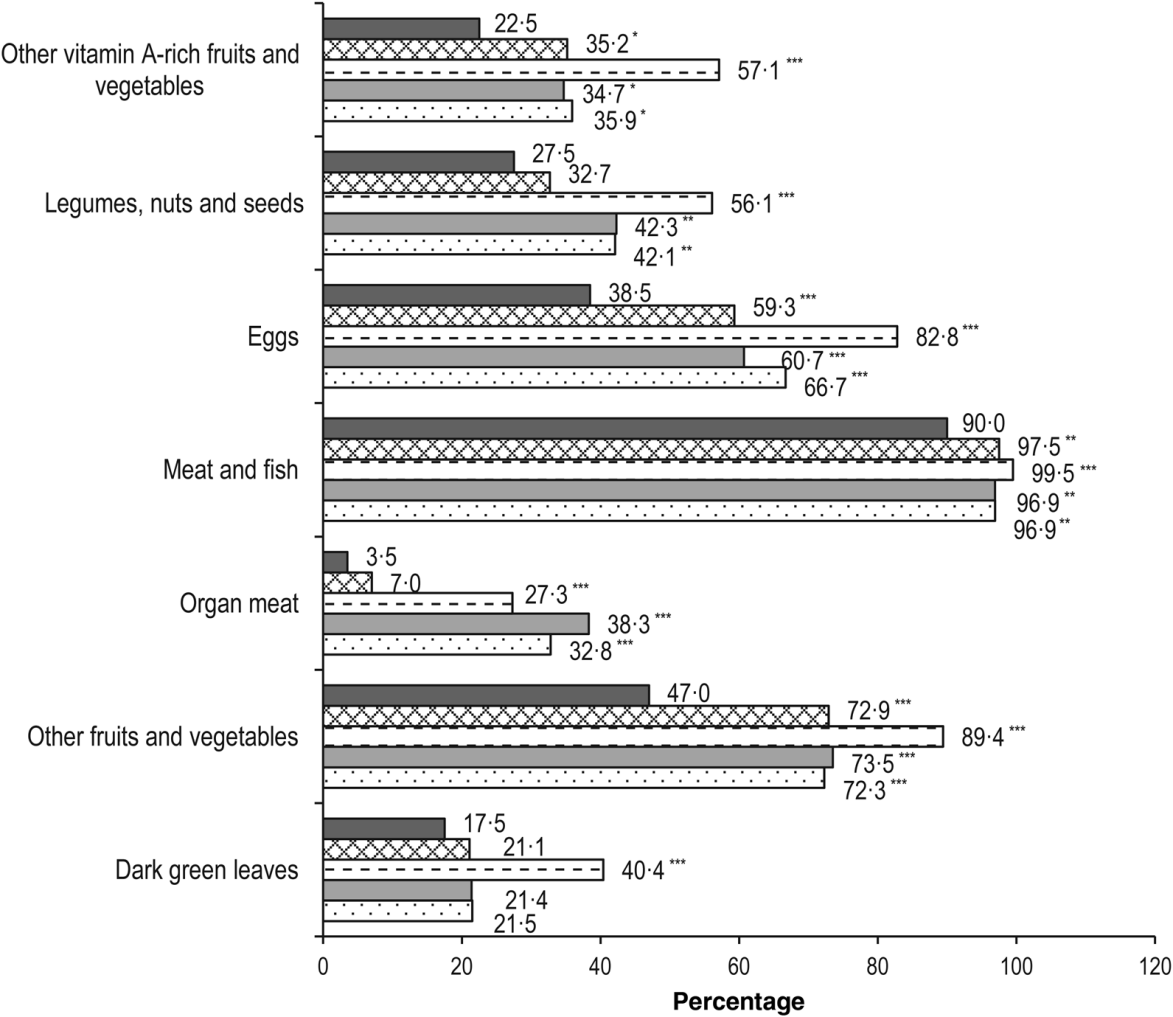
Food group consumption at baseline (■) and each post-baseline visit: week 4 (×); week 16 (=); week 32 (▒); week 48 (░). *P* values are from McNemar's test and controlled for multiple comparisons using step-down Bonferroni adjustment: * *P* < 0·05, ** *P* < 0·01, *** *P* ≤ 0·0001.

In terms of food group consumption patterns, starchy staples were shown to be always included in the child's diet throughout the study period of 48 weeks, except for one child (0·5 %) at the baseline visit, who did not report consuming starchy staples (data not shown). Because all children were reported to consume at least 85 % of the recommended amount of ONS over the study period, the percentage of children that consumed milk and milk products increased from 22·5 % at baseline to 100 % at each of the post-baseline time points (data not shown). As shown in Fig. 2, the number of children who consumed other vitamin A-rich fruits and vegetables, other fruits and vegetables, meat and fish, and eggs was significantly higher than baseline within 4 weeks of intervention (*P* = 0·0105, <0·0001, 0·0043, and <0·0001, respectively) and remained significantly higher through the rest of the study period. For other food groups including organ meat, and legumes, nuts and seeds, the number of children consuming these food groups was also significantly higher than at baseline from week 16 onwards (*P* < 0·0001 and *P* < 0·05, respectively), whereas it reached significance at week 16 for dark green leafy vegetables (*P* < 0·0001).

**Fig. 3.**
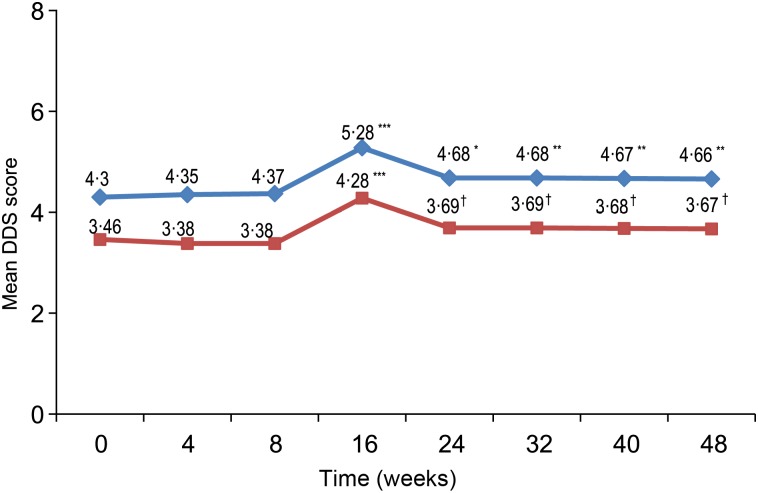
Mean dietary diversity score (DDS) at baseline and each post-baseline visit. 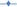, With milk or milk products/oral nutritional supplement (ONS); 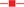, without milk or milk products/ONS. *P* values are from the signed-rank test and controlled for multiple comparisons using step-down Bonferroni adjustment: * *P* = 0·0011, ** *P* = 0·008, *** *P* ≤ 0·0001, † *P* = 0·068.

**Fig. 4.**
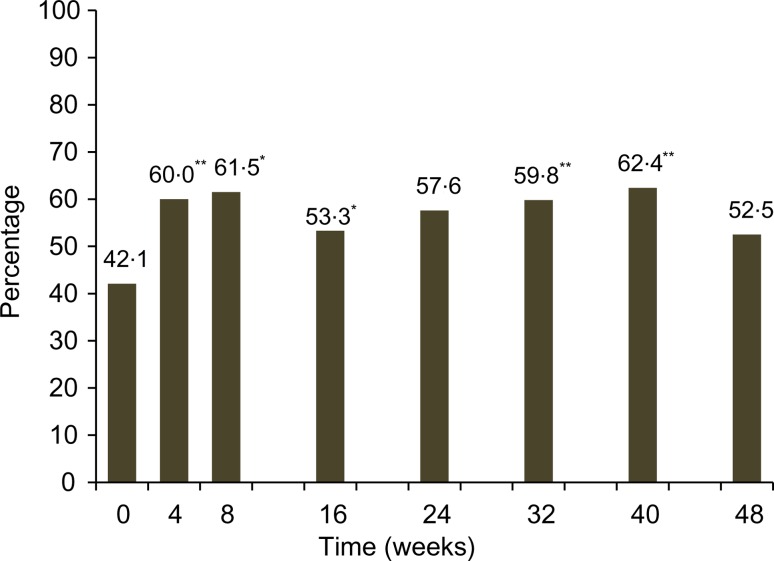
Percentage of children consuming a diet following the recommended macronutrient distribution at baseline and each post-baseline visit. *P* values are from McNemar's test and controlled for multiple comparisons using step-down Bonferroni adjustment: * *P* < 0·01, ** *P* < 0·001.

Fig. 3 shows the mean DDS at baseline and each post-baseline visit with and without milk and milk product, and ONS consumption. There was an increasing trend of consuming more variety of food groups from week 16 onwards. The mean DDS reached significance at week 16 (*P* < 0·0001) and remained significant (*P* < 0·05) or a trend for the rest of the study (*P* = 0·0680) before and after excluding milk and ONS consumption, respectively. This is supported by a significant increasing relationship for energy (*P* < 0·0001), protein (*P* = 0·0021), carbohydrate (*P* = 0·0258), fat (*P* < 0·0001) and Ca (*P* = 0·0071) intakes from foods alone over the period of 48 weeks (data not shown). There was also a significant increase in the percentage of children who consumed diets meeting the recommendation of macronutrients at all post-baseline visits, except for weeks 24 and 48 (Fig. 4).

### Effects of nutrient intakes from foods and oral nutritional supplementation on catch-up growth and growth maintenance over the study period

Tables 1 and 2 show the GEE models of sociodemographic and nutritional factors associated with WHP and HAP development, respectively, over a 1-year period. A significant increase in WHP was observed after 4 weeks of intervention; however, the amount of WHP increase over time is quite stable, varying between 13·7 and 16·1 units at each post-baseline time point compared with baseline (*P* < 0·0001; Table 1). Parental employment status was negatively related to increased WHP when compared with unemployment status (*P* < 0·05). None of the studied nutrients was significantly related to WHP or HAP values. Additional energy intake of 100 kcal was significantly associated with a 0·25 percentile increase in WHP (*P* = 0·0027). Increases in HAP compared with baseline reached significance at week 16 and remained significant through week 48, with percentile increases ranging from 2·16 units at week 16 to 3·99 units at week 48 (*P* < 0·01; Table 2). Females were associated with significantly lower WHP than males, but there was no significant association in HAP among sexes. Higher parental education was positively associated with HAP (estimate: 4·95; *P* = 0·0348). In addition, a 1 % increase in ONS compliance was associated with a 1·25 percentile increase in HAP over the study period (*P* = 0·0221).

**Table 1.** Generalised estimating equation (GEE) model of factors associated with weight-for-height percentile over the 1-year period (Estimates and 95 % confidence intervals)

Factors	Estimate	95% CI	P*
Intercept	14·37	−7·85, 36·59	0·2050
Visit			
Baseline	0		
Week 4	13·71	10·69, 16·73	<0·0001
Week 8	15·18	11·80, 18·56	<0·0001
Week 16	16·11	12·25, 19·97	<0·0001
Week 24	16·01	12·29, 19·73	<0·0001
Week 32	15·46	11·03, 19·89	<0·0001
Week 40	15·93	11·49, 20·37	<0·0001
Week 48	15·00	10·60, 19·40	<0·0001
Age at baseline (months)	0·18	−0·35, 0·71	0·5091
Sex			
Male	0		
Female	−2·19	−4·24, −0·14	0·0365
Highest parental occupation			
Unemployed	0		
Self-employed	−8·64	−15·86, −1·42	0·0191
Government or military	−14·21	−22·65, −5·77	0·0010
Private sector	−7·96	−13·38, −2·55	0·0039
Visit × sex interaction			
Baseline × male	0		
Baseline × female	0		
Week 4 × male	0		
Week 4 × female	−4·42	−8·31, −0·53	0·0260
Week 8 × male	0		
Week 8 × female	−3·20	−7·61, 1·21	0·1546
Week 16 × male	0		
Week 16 × female	−3·34	−8·09, 1·42	0·1690
Week 24 × male	0		
Week 24 × female	−4·31	−8·90, 0·28	0·0658
Week 32 × male	0		
Week 32 × female	−6·41	−11·77, −1·04	0·0192
Week 40 × male	0		
Week 40 × female	−4·92	−10·62, 0·79	0·0912
Week 48 × male	0		
Week 48 × female	−4·58	−10·37, 1·20	0·1205
Energy intake (100 kcal)	0·25	0·09, 0·41	0·0027

* *P* value is from the GEE analysis.

**Table 2.** Generalised estimating equation (GEE) model of factors associated with height-for-age percentile over the 1-year period (Estimates and 95 % confidence intervals)

Factors	Estimate	95% CI	P*
Intercept	−82·75	−186·54, 21·05	0·1182
Visit			
Baseline	0		
Week 4	0·12	−0·56, 0·80	0·7246
Week 8	1·03	−0·11, 2·18	0·0776
Week 16	2·16	0·70, 3·63	0·0038
Week 24	2·88	1·31, 4·45	0·0003
Week 32	3·43	1·73, 5·12	<0·0001
Week 40	3·74	1·95, 5·54	<0·0001
Week 48	3·99	1·82, 6·17	0·0003
Age at baseline (months)	−0·72	−1·34, −0·11	0·0214
Sex			
Male	0		
Female	−0·90	−5·68, 3·89	0·7130
Highest parental education			
High school	0		
College/university or higher	4·95	0·35, 9·54	0·0348
Visit × sex interaction			
Baseline × male	0		
Baseline × female	0		
Week 4 × male	0		
Week 4 × female	−0·39	−1·31, 0·53	0·4080
Week 8 × male	0		
Week 8 × female	−0·88	−2·33, 0·58	0·2372
Week 16 × male	0		
Week 16 × female	−1·59	−3·61, 0·42	0·1217
Week 24 × male	0		
Week 24 × female	−1·56	−3·69, 0·57	0·1505
Week 32 × male	0		
Week 32 × female	−1·78	−3·95, 0·40	0·1089
Week 40 × male	0		
Week 40 × female	−2·01	−4·27, 0·25	0·0816
Week 48 × male	0		
Week 48 × female	−2·44	−5·09, 0·21	0·0714
Overall ONS compliance	1·25	0·18, 2·32	0·0221

ONS, oral nutritional supplementation.

* *P* value is from the GEE analysis.

## Discussion

This study showed that long-term nutritional intervention consisting of initial dietary counselling and continuous ONS not only improved but also sustained adequate intake of nutrients that are limited in Filipino children's diets. Total energy intake from foods and ONS and compliance with ONS were associated with an improvement of WHP and HAP over time, respectively, after controlling for sociodemographic factors. Furthermore, the nutritional intervention was associated with increased DDS and increased consumption of nutrients from foods during the course of the intervention.

Nutritional adequacy is essential for promoting growth and health in children^(^^1^^)^. However, Filipino children are reported to have diets low in nutrient densities, which do not meet the WHO recommendation^(^^2^^)^. Data from a nationwide study indicate that one-quarter to one-third of Filipino children aged 6 months to 5 years consumed a diet deficient in energy and nutrients including protein, vitamin A, vitamin C, Fe and Ca^(^^23^^)^. In the present study, the percentage of children consuming adequate energy and nutrients was higher than that reported from the national nutrition survey. A plausible explanation is that the children in this study belonged to the middle class as gleaned by the higher education levels of their parents such as having high school attainment at least. Still, about two-thirds of these children did not consume adequate energy, and 40 to 50 % of them were at risk of vitamins A and C, and Fe deficiencies because of inadequate nutrient levels in their diets before receiving the intervention. The percentage of children who achieved energy and nutritional adequacy was significantly increased within 4 weeks of the intervention and reached nearly 100 % for the assessed nutrients based on the IDES for the remaining study period (Fig. 1). These results are in accordance with previous studies that reported reducing the risk of deficiencies of important nutrients for growth and immune functions including α-linolenic acid, Fe, vitamin C and vitamin D in children consuming non-fortified milk^(^^24^,^25^^)^. The findings from the present study suggest that long-term nutritional intervention using ONS may help improve and sustain nutritional adequacy over the study period of 48 weeks^(^^11^^)^.

Growth during early childhood is largely influenced by environmental factors including nutritional and socio-economic factors although genetic endowment also plays a key role^(^^26^^–^^28^^)^. In this study, both sociodemographic and nutritional factors like parental occupation and total energy intake from foods and ONS were associated with WHP development over time. Higher total energy intake from foods and ONS was significantly associated with greater increase in WHP over the study period. Furthermore, the amount of percentile gain achieved at week 4 was greatest between any two subsequent post-baseline time points, suggesting an impact of the intervention on adequate energy intake that enables catch-up growth. After week 4, the amount of WHP gain from baseline to any other post-baseline time point was similar, indicating growth maintenance. While maternal employment was shown to be a protective factor for wasting in a cohort study of Filipino young children in urban barangays^(^^29^^)^, we observed that parental employment status was negatively associated with WHP gain over time in the present study (Table 1). There does not seem to be a reasonable explanation for this observation, except perhaps to surmise that with both parents being employed, the quality and quantity of time spent in taking care of their children may be compromised. This may require further research; however, the findings suggest that nutritional intervention should take these factors into consideration as they may affect the success of the intervention.

Higher parental education level and higher compliance with ONS were shown to be associated with HAP gain over time in the present study (Table 2). Higher parental education levels were previously reported to be associated with protective caregiving behaviours, thus lowering the risk of stunting in a cohort study of Filipino young children^(^^29^^)^. Therefore, additional counselling techniques and closer monitoring for parents with lower educational levels may be needed to ensure success of nutritional intervention for improving height. Previous studies reported positive associations between specific nutrient intake such as vitamin A, Zn or protein with ponderal and linear growth^(^^30^^–^^32^^)^, suggesting that intake of certain nutrients may be specifically important to promote growth. In this study, we investigated the impact of nutritional intakes and dietary diversity on longitudinal linear and proportional growth, controlling for sociodemographic factors. None of the specific nutrients from the diet was shown to be associated with growth outcomes over time. However, a higher compliance with ONS was significantly associated with linear growth. Because all children in this study were reported to consume at least 85 % of the recommended intake of ONS, higher compliance indicates greater intake of essential nutrients for growth provided by ONS. According to Golden^(^^5^^)^, there are about forty nutrients essential for health, and their deficiencies will negatively affect a child's growth and disease resistance or recovery from illness. Therefore, these findings suggest that it is unlikely that a single nutrient intervention would play a more critical role than the others in promoting growth. This is also supported by the findings from a recent meta-analysis by Stammers *et al*.^(^^33^^)^ showing that Zn supplementation alone may have only a limited effect on both weight and height in children aged 1–8 years. In the present study, children achieved a significant increase in HAP at week 16 with a subsequent steady, albeit small, increase for the remaining study period (Table 2). As noted by Golden^(^^5^^)^, stunted children will need to have a sustained increase in dietary nutrient quality to allow for increased rates of height gain to be maintained over a prolonged period. Height gain in children in this study was modest, when compared with that in moderately undernourished children aged 2–5 years, convalescing from shigellosis^(^^34^^)^. These children demonstrated an increase in height almost twice the normal growth rate, using the WHO Growth Standards, over a 21-d period of treatment with a high-protein diet^(^^34^^)^. This amount of height gain is reasonable because the intervention in this study used the recommended intake for normal children as the nutritional target, and also children who were at nutritional risk only and beyond the age group with high potential to catch-up in height above 3 years. The ONS used in this study fulfils the recommended nutritional characteristics in terms of energy density and essential growth nutrients of diet appropriate for stunted children^(^^8^^)^. Therefore, this study found that ONS is effective in improving and sustaining nutrient density and adequacy which helped promote and sustain height growth over the intervention period.

Children are recommended to consume a variety of food groups, particularly meat, poultry, fish, eggs, fruits and vegetables, for improving the nutrient adequacy of the diet to meet their requirements, in addition to milk and milk products^(^^35^^)^. In this study, although ONS significantly contributed to nutrient adequacy directly, we also observed an improving trend in food diversity in the children's diets as measured by the DDS which reached significance at week 16, and remained significant or indicated a trend afterwards before and after excluding milk and milk products. In addition, the number of children who reported consuming important food groups such as meat and fish, eggs, organ meat, fruits and vegetables increased significantly from week 4 onwards when compared with baseline. The positive findings on healthier eating habits observed as early as at week 4 of intervention suggest the initial impact of dietary counselling given to the parents during the first 8 weeks on improving dietary variety similar to other intervention studies using dietary counselling for improving food diversity in children^(^^36^^)^. However, the improved DDS continued after week 16 is consistent with other previously reported positive outcomes of using long-term ONS in the nutritional intervention such as maintaining nutritional status with balanced growth, improving physical activity and appetite scores as well as having fewer number of sick days for the remaining period^(^^11^^)^. A greater diversity usually results in diets of higher absolute levels of energy and nutrients due to an increase in food intake^(^^37^^)^. In the present study, along with an increase in dietary diversity, the energy, macronutrient and Ca intakes from foods only, after excluding the milk consumption at baseline and ONS consumption at post-baseline time points, significantly increased or indicated a trend over the study period. Additionally, the percentage of children consuming diets following macronutrient distribution recommendation increased from baseline to most of the post-baseline time points. It should be noted that the ONS contains the energy percentage coming from carbohydrate, fat and protein that is very close to the recommendation for Filipino children, namely, containing 12, 35 and 53 % energy from protein, fat and carbohydrate, respectively. These findings suggest that children had increased consumption of family foods with greater variety of foods compared with baseline in a more balanced manner. This healthier eating habit along with previously reported positive outcomes on growth and health outcomes^(^^11^^)^ reflect an improvement in the overall health of children receiving long-term nutritional intervention using ONS. Thus, the study findings suggest continuous consumption of ONS providing 30 % of the energy requirement did not replace the intake of foods from the regular diet while it had positive impact on nutritional adequacy and growth in children at risk of undernutrition.

This study was a single-arm clinical trial. As such there are some limitations that have been discussed previously^(^^11^^)^. Because of the lack of a control group in the study design and the high compliance of ONS consumption in these children there are some caveats in the causal assessment of the effect of the intervention on the interested outcomes such as the improving DDS. This positive outcome deserves further investigation in a placebo-controlled clinical trial although the improved food diversity was consistent with other benefits on growth and health observed in this child population; thus, it supports the efficacy of the nutritional intervention using long-term ONS. The use of single 24-h recall at baseline *v*. two 24-h recalls at post-baseline visits may result in less comparable dietary intake data between baseline and post-baseline visits. In addition, as suggested by other authors, subsequent dietary recalls may be affected by a ‘learning effect’ where respondents overestimate food consumption on the first administration and become more familiar with the recall procedure leading to more accurate estimates of the food intake on subsequent administration^(^^38^^,^^39^^)^. However, the post-baseline improvement in dietary intake is in accordance with the improvement in growth and other observed health benefits, suggesting that a single 24-h recall at baseline provided a relative measure of dietary intake representing the true usual dietary intake values as two 24-h recalls, and the possible learning effect may be small. Moreover, while the use of a portion size such as 10 g minimum food group may help further refine DDS and improve the correlations between DDS and nutrient adequacy^(^^40^^)^, we did not include a portion size requirement in the assessment of DDS. Nonetheless, the intention of this assessment was to investigate whether use of ONS interferes with the consumption of normal foods rather than the correlations between DDS and nutrient adequacy. Also, the assessment of ONS compliance using parental self-reporting may be subject to bias. Another limitation is that there were a number of important nutrients for growth that were not available due to the incompleteness of the food composition database. This may limit a comprehensive assessment of the relationship between nutrient intake and growth. In addition, the Goldberg cut-offs based on an assumed light physical activity level in adults may overestimate misreporting in children when the over-reporting problem seems to be more noticeable when coming closer to the end of the study. It is possibly a result of ‘trial effect’ in which parents or caregivers were receiving extra care from the healthcare professionals and were under regular follow-up which was previously suggested to increase ONS compliance^(^^41^^)^. Nevertheless, nutritional status was comparable between normal- and misreporters for most of the study time points, suggesting that using this method for identifying misreporting may help minimise the impact of overestimation of nutritional intake. Furthermore, the overestimation of misreporting may also underestimate rather than overestimate the assessment of nutritional adequacy in these children.

In conclusion, the long-term nutritional intervention using initial dietary counselling and continuous ONS has been shown to promote and sustain adequate intake of nutrients that are limited in Filipino children's diets without displacing normal family foods. In fact, the results showed that ONS use was associated with improved food diversity. The study further supports the effectiveness of supplementation of multiple nutrients including macro- and micronutrients, rather than a single nutrient in meeting the increased nutritional needs for catch-up growth and growth maintenance in both weight and height. Thus such multiple-nutrient intervention may be beneficial in preventing and improving wasting and stunting in children at risk of undernutrition. The results of this study further suggest that ONS may be a useful supportive tool in helping nutritionally at-risk children to achieve nutritional adequacy and healthier growth.
